# Glossary of Terms Applied in Electrical Science

**Published:** 1890-12

**Authors:** 


### EDITORIAL.

### Glossary of Terms Applied in Electrical Science.

*Ampere* (The). Is the unit of strength of current.

*Anode.* The positive pole of a battery.

*Cathode.* The negative pole of a battery.

*Conductor.* The wire or cord through which the electricity is conveyed from the battery to the patient.

*Constant Battery.* One capable of giving a continuous current with unvarying constancy.

*Continuous Current.* Term applied to the galvanic current in opposition to the electro-magnetic.

*Current Selector.* A contrivance for enabling the operator to use either the galvanic, or primary and secondary Faradic.

*Commutator.* An arrangement for reversing the current.

*Electrode.* An instrument for the application of electricity.

*Electrometer.* An instrument by which the force of an electric current is measured.

*Element.* (In electricity). A voltaic couple, pair, or cell.

*Electro Motive Force.* Which is the force that moves electricity, is usually written E. M. F.

*Electrolysis.* Electro-chemical decomposition.

*Electroscope.* Same as Galvanoscope.

*Faradic Current.* The induced current. The term is applied both to the electro-magnetic and magneto-electric currents, since they were both discovered by Faraday; called also secondary, interrupted, induced, inductive, to and fro, indirect, electro-magnetic, and magneto-electric; the term Faradic is more universally used.

*Faridization.* The use of the Faradic current.

*Galvanoscope.* (Galvanometer). An instrument by which we detect small quantities of electricity, and distinguish between positive and negative.

*Hydrostat.* An arrangement to prevent accidental escape of battery fluid from the cells.

*Interrupted Current.* Broken, intermitted. The Faradic is necessarily interrupted by the apparatus that generates it; the galvanic may be continuous or interrupted.

*Insulator.* A poor conductor of electricity.

*Insulated.* Placed on non-conducting supports, or covered with non-conducting substances.

*Labile Current.* An application in which one or both of the electrodes are moved or glided over the surface.

*Magnets.* Substances that have the property of attracting iron.

*Negative Pole.* Where the current passes out; called also, zinc-pole, or cathode.

*Non-Conductor.* Glass, resin, vulcanite, and all substances that do not transmit electricity freely are thus called.

*Ohm* (The). Is the unit of resistance. (R).

*Ohm's Law.* This important law may be stated as follows: The strength of the current varies directly as the electro-motive force, and inversely as the total resistance. Or, the number of amperes of current flowing through a circuit is equal to the number of volts of electro-motive force divided by the number of Ohms of resistance in the electric current.—E–R equals C.

*Poles.* Points where magnetism is concentrated, or where the electric current passes in and out.

*Primary, or Inducing Current.* The current that passes through the inner coil of wire in a helix, and that induces a current on the coil that surrounds it. Used erroneously as synonymous with galvanic, or constant current.

*Rheotome.* A current breaker.

*Rheotrope.* Current reverser.

*Secondary Currents.* That which is supplied by the outer coil of the electro-magnetic machine.

*Static Electricity.* Electricity generated by friction.

*Volt* (The). Is the unit of electro-motive force [E].

W. H. W.

---

Dr. Walter Rivington says that to improve the medical profession in Great Britain it is necessary to diminish the over-crowding, and the too free use of alcohol.

